# Characterization of prokaryotic communities in Guánica State dry forest caves using 16S ribosomal RNA gene amplicon sequencing

**DOI:** 10.1128/mra.00646-26

**Published:** 2026-09-14

**Authors:** Camila M. Martínez-Llaurador, Isabel Aguirre-Ortiz, Francisco J. Román-Soto, Peter A. Camacho-Nazario, Naomy M. Chaparro-Mata, Tesia Lin, Josué Rodríguez-Ramos, Ángel M. Nieves-Rivera, Miguel J. Martínez-González, Miguel Canals-Mora, Carlos Rios-Velazquez

**Affiliations:** 1 Microbial Biotechnology and Bioprospecting Laboratory, Biology Department, University of Puerto Rico 16146 , Mayagüez, Puerto Rico, USA; 2 Industrial Biotechnology Program, University of Puerto Rico 16146 , Mayagüez, Puerto Rico, USA; 3 Pacific Northwest National Laboratory, Biological Sciences Division 536904 , Richland, Washington, USA; 4 Grupo Espeleológico del Oeste, Inc. (GEO), Mayagüez, Puerto Rico, USA; Indiana University Bloomington, Bloomington, Indiana, USA

**Keywords:** 16S rRNA gene amplicon, caves, Guánica State Forest, Puerto Rico, microbial diversity

## Abstract

Prokaryotic diversity across four caves and a guano-enriched pond (mud) in Guánica State Dry Forest was characterized using 16S ribosomal RNA gene amplicon sequencing. Microbial profiling varies across the limestone rock formations and dry cave environment, revealing distinct subterranean prokaryotic composition.

## ANNOUNCEMENT

Caves exhibit challenging environmental conditions, including darkness, high humidity, and low nutrient availability (1, 2), yet they sustain diverse microbial communities involved in biogeochemical cycling and the production of bioactive compounds (3, 4). Culture-independent approaches provide broader insight into cave microbial diversity because many cave microorganisms are difficult to cultivate. Guánica State Forest is characterized by a subtropical dry climate, drought-adapted biodiversity, and extensive limestone cave systems (5, 6). Cueva de los Murciélagos is an extensive cave system with multiple entrances, including Cueva Carita, which provides access to a guano-enriched pond-mud environment; hydrological and geological barriers create distinct microenvironments within the cave network (7). Sample details are presented in Table 1. Thirty grams of superficial bat guano-enriched soil or pond mud were collected in sterile Whirl-Pak bags and refrigerated at 4°C until DNA extraction. Genomic DNA was extracted from 10 g of each sample suspended in 20 mL Z-buffer containing 0.02 g lysozyme. Cells were disrupted by repeated freeze-thaw cycles, followed by lysis with 2.25 mL sodium dodecyl sulfate and 1.5 mL guanidinium isothiocyanate at 65°C for 2 h. Lysates were centrifuged, extracted with an equal volume of chloroform, and precipitated with 1 volume of isopropanol at −20°C overnight. DNA pellets were centrifuged, washed with 70% ethanol, air-dried, resuspended with T_10_E_10_, and stored at −80°C (8–11). DNA fragments up to 25 kb were purified by gel electrophoresis, excised, and digested with β-Agarase I according to the manufacturer’s protocol (12). DNA quality and quantity were assessed using a BioPhotometer Plus UV/Vis Photometer (Eppendorf). Purified DNA was submitted to Molecular Research DNA Laboratory (MR DNA, Shallowater, TX, USA) for bTEFAP Illumina 20K sequencing targeting the 16S ribosomal RNA (rRNA) V3–V4 gene region. Amplification used primers 341F (5′-CCTACGGGNGGCWGCAG-3′) and 785R (5′-GACTACHVGGGTATCTAATCC-3′) (13), sample-specific barcodes, and HotStarTaq Plus Master Mix Kit (Qiagen, USA). PCR consisted of 95°C for 5 min; 30 cycles of 95°C for 30 s, 53°C for 40 s, and 72°C for 1 min; and 72°C for 10 min. Sequencing was performed on an Illumina MiSeq using 2 × 300 bp paired-end reads. Demultiplexed reads were processed in QIIME (2026.1) using a paired-end 16S rRNA gene amplicon pipeline (14). Quality filtering, denoising, chimera removal, and ASV inference were performed with DADA2 version 2026.1.0 using a 25-base trim from the 5′ end of reverse reads and a maximum expected error threshold of 3 for both forward and reverse reads (15). The DADA2 assignTaxonomy and assignSpecies functions were not used for taxonomic assignments. Reads were clustered at a 100% similarity, and default parameters were used unless otherwise specified. Taxonomy was assigned using the classify-sklearn method with the SILVA v138 99% Naïve Bayes classifier. Low chimeric read retention may reflect reduced overlap and increased read loss during merging and chimera filtering of the longer 16S V3–V4 amplicons using 2 × 300 bp reads. Nevertheless, retained reads provided a general profile of prokaryotic diversity. Proteobacteria was the most abundant phylum, followed by Actinobacteriota, Bacteroidota, Acidobacteriota, and Chloroflexi (Fig. 1).

**TABLE 1:** Sampling sites, SRA accessions, barcode sequences, and sequencing output statistics for 16S rRNA gene amplicon analysis

Sampled caves	Sample ID	Coordinates	Collection date	SRA accession	Barcode sequence	Raw input reads	Percentage passed filter	Denoised	% of non-chimeric reads
Cueva de los Murciélagos	MUR	17° 57′16″ N, 66° 50′54″ W	17/05/2025	SRX33109040	CCTCGCAACC	748,207	94.87	685,442	11.36
Cueva Carita	CRT	17° 57′16″ N, 66° 50′54″ W	17/05/2025	SRX33109041	GTATAGCTGT	580,999	95.58	501,137	24.02
Cueva Geña La Negra	GEN	17° 57′19″ N, 66° 51′10″ W	28/06/2025	SRX33109042	GCTACATTAG	630,023	95.51	596,350	37.32
Cueva Del Negro	NEG	17° 58′11″ N, 66° 52′35″ W	28/06/2025	SRX33109043	TACGAATCTT	593,667	95.77	520,964	23.44
Lago de Guano	GUA	17° 57′37″ N, 66° 51′08″ W	17/05/2025	SRX33109044	GAGTGTGCCG	506,980	95.16	427,575	27.69

**Fig 1:**
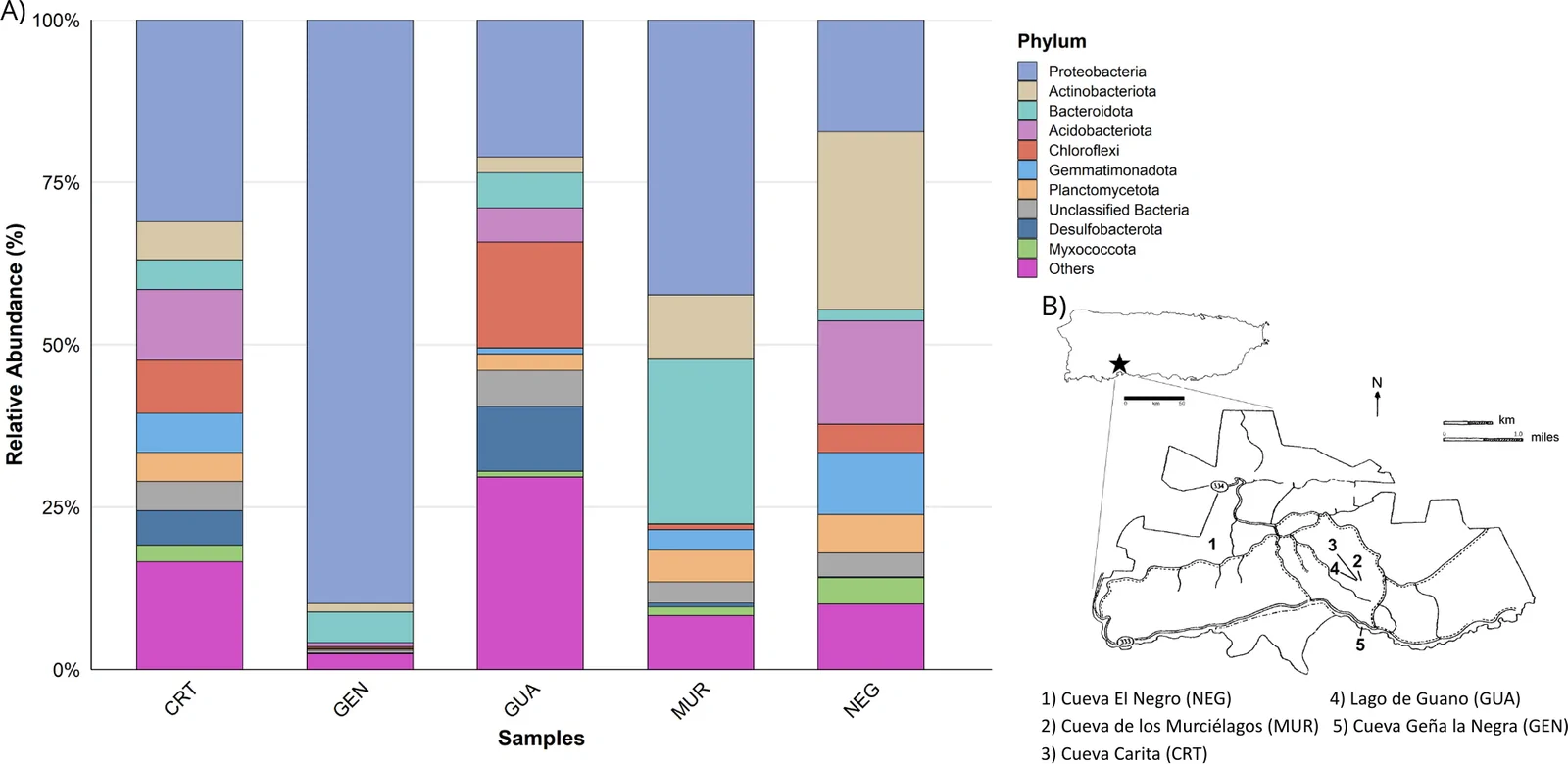
Relative abundance of prokaryotic communities across four caves and one pond mud in the Guánica State Forest. (**A**) Taxa bar plots illustrate the relative abundance of microbial phyla across samples, with phyla representing less than 5% of the total abundance grouped into a single category labeled “others.” (**B**) Location of caves and guano-enriched lake in the Guánica State Forest and its location in Puerto Rico. Ángel M. Nieves Rivera generated the map.

## Data Availability

The raw data from this project has been deposited in the National Center for Biotechnology Information under the BioProject accession number PRJNA1457879. The samples were distributed as follows: Cueva Carita (CRT) (Sequence Read Archive [SRA] accession number SRX33109041), Cueva Geña La Negra (GEN) (SRA accession number SRX33109042), Lago de Guano (GUA) (SRA accession number SRX33109044), Cueva de los Murciélagos (MUR) (SRA accession number SRX33109040), Cueva del Negro (NEG) (SRA accession number SRX33109043).
